# Benefits of linking civil registration and vital statistics with identity management systems for measuring and achieving Sustainable Development Goal 3 indicators

**DOI:** 10.1186/s41043-019-0178-0

**Published:** 2019-10-18

**Authors:** Samuel Mills, Jane Kim Lee, Bahie Mary Rassekh

**Affiliations:** World Bank Group, 1818 H Street, NW, Washington, DC, 20433 USA

**Keywords:** Civil registration, Vital statistics, National identification, Sustainable Development Goals, Unique identification number, Birth registration, Population register, Policy, Maternal mortality ratio, Under-five mortality, Universal health coverage

## Abstract

A complete civil registration and vital statistics system is the best source of data for measuring most of the Sustainable Development Goal 3 indicators. However, civil registration does not include migration data, which are necessary for calculating the actual number of people living in a given area and their characteristics such as age and sex. This information is needed to facilitate planning, for example, for school places, health care, infrastructure, etc. It is also needed as the denominator for the calculation of a range of health and socioeconomic indicators. Obtaining and using these data can be particularly beneficial for measuring and achieving universal health coverage (Target 3.8), because civil registration can help to identify persons in need of health care and enable decision-makers to plan for the delivery of essential services to all persons in the country, including the most disadvantaged populations. By assigning unique identification numbers to individuals, for example, at birth registration, then using these numbers to link the individuals’ data from civil registration, national identification, and other functional registers, including registers for migration and health care, more accurate and disaggregated population values can be obtained. This is also a key to improving the effectiveness of and access to social services such as education, health, social welfare, and financial services. When civil registration system in a country is linked with its national identification system, it benefits both the government and its citizens. For the government, having reliable and up-to-date vital events information on its citizens supports making informed program and policy decisions, ensuring the accurate use of funds and monitoring of development programs at all levels. For individuals, it makes it easier to prove one’s identity and the occurrence of vital events to claim public services such as survivor benefits or child grants.

## Main text

Civil registration is the universal, continuous, permanent, and compulsory recording of vital events (for example, birth, death, marriage, divorce, and adoption) in accordance with the legal requirements of each country [1]. Along with the characteristics of vital events collected for statistical purposes, an individual’s biographical information (such as name, date of birth, place of birth, and names of the parents) is recorded through civil registration to establish a legal identity. National identification systems typically add other attributes of the individual, such as a unique identification number (UIN), photograph, signature, and biometrics (for example, fingerprint, facial recognition, hand geometry, voice recognition, iris scan, and retinal scan), which can be used by individuals to prove their identity for a wide range of activities, including voting, opening a bank account, buying or inheriting property, paying taxes, enrolling in a health insurance plan, traveling, and qualifying for a cash transfer. Despite these crucial roles that civil registration and national identification systems play, these systems are neither functional nor linked to each other in many low- and middle-income countries [2].

## Civil registration and vital statistics (CRVS) is the optimal source of data for calculating most Sustainable Development Goal (SDG) 3 indicators

CRVS is the most reliable source of data for calculating 12 of the 13 targets of SDG 3 [3]. For the health sector, CRVS provides routine and disaggregated population data for monitoring national development plans for health as well as progress toward SDG 3: *Ensure healthy lives and promote well-being for all at all ages*. Some SDG 3 indicators require CRVS data for both the numerator and denominator values. For example, Indicator 3.1.1: Maternal Mortality Ratio uses the number of maternal deaths for the numerator and the number of live births during the same time period for the denominator, and CRVS is the best source for both these numbers through birth and death registration. For other SDG 3 indicators, the numerator values may be best collected using other methods, such as surveillance systems or incidence-testing in population surveys (for example, incidence of HIV [3.3.1], tuberculosis [3.3.2], malaria [3.3.3], and hepatitis [3.3.4]). However, the number for the denominator (per 1,000 or 100,000 population) requires the total number of the population (obtained from CRVS records) to complete the calculation.

## Why is CRVS currently not used to monitor most SDG 3 indicators?

It is important to understand the drawbacks of having insufficient CRVS systems. Most low- and middle-income countries that do not have complete CRVS systems have used data from other sources, typically population-based surveys, to measure SDG 3 indicators. These sources have provided good estimates but are only conducted every 3 to 5 years. The statistics derived from population surveys are affected by sampling errors, recall bias, and are usually derived using indirect techniques based on assumptions, hypothesis, and models; therefore, high-quality civil registration data, when complete, are more reliable. Also, although these surveys provide national-level data, they do not provide disaggregated data that are necessary for planning and monitoring health programs at lower administrative levels. For example, population-based surveys do not provide the requisite data to inform district health management teams about progress in increasing the proportion of births that are attended by skilled health personnel (3.1.2) or the proportion of institutional births. Thus, district health management teams tend to rely on health information systems for the numerator values and on population projections from censuses for the denominator values; however, these population projections may be less accurate during the expected 10-year intercensal period.

Similarly, estimates of the maternal mortality ratio (MMR) or under-five mortality for most countries are generated using data from population-based surveys. Although the sources of data and methods of estimating MMR have improved over time, the United Nations Maternal Mortality Estimation Inter-Agency Group has recommended that countries “establish well-functioning civil registration systems with accurate attribution of cause of death,” because they are the best source of data for directly computing and monitoring the MMR and causes of maternal mortality [4–6]. Although this recommendation has been made repeatedly over the last two decades, CRVS systems in many countries are under resourced, lack adequate political commitment, and in need of significant strengthening to provide the necessary data to prepare these estimates. Ascertaining cause of death is important and can be done using medical certification for deaths in hospitals and verbal autopsy for deaths in the community.

## Assigning UIN at birth registration allows linkage of civil registration and national identification systems

Target 16.9 of the Sustainable Development Goals (SDG)—*By 2030*, *provide legal identity for all*, *including birth registration*—presents an opportunity for countries to link civil registration and national identification systems, which has benefits for multiple sectors. Linking the two, particularly by assigning a UIN at birth, is key to improving access to social services such as education, health, social welfare, and financial services (for example, credits or inheritances). Ensuring the visibility and accurate identification of all residents, including refugees and stateless persons, facilitates the provision of targeted services to the poor, such as essential and high-quality health services and social safety net programs, ultimately contributing to achieving universal health coverage (UHC, SDG target 3.8) and substantial coverage of the poor and the vulnerable through social protection systems (SDG target 1.3).

The data link between civil registration and national identification systems is possible through the UIN assigned to each individual at birth. This same UIN is used later in life on the national identity card, depending on the laws of the country, as well as on various legal and other documents that a person receives during his or her lifetime. For example, if an individual’s birth certificate, marriage certificate, and national identification card are all associated with the person’s UIN, then that number can serve as a linkage between the databases belonging to different ministries and agencies that oversee each vital event.

In some countries, a birth registration number (BRN) is assigned at birth registration to uniquely identify the birth record, and separately, a UIN is assigned when the child reaches the age of obtaining a national identity card. In this case, each individual has two different numbers, one in the civil registration database and another in the national identification database. The World Bank proposed three options for linking BRN and UIN [7].

i. If the UIN is generated by the national identification system, during birth registration, the civil registration system can also generate a BRN but request the national identification system to generate a UIN. This UIN is linked to the BRN in the civil registration database. In this way, a UIN is assigned to each birth record and is subsequently linked with other vital events such as marriage, deaths, and so forth, for the same individual.
ii. If the UIN is generated by the civil registration system, then there is no need to generate a separate BRN and the UIN is transmitted to the national identification system for use later for the national identity card, and so forth.
iii. If the same department or agency administers both civil registration and national identification, then the UIN that is generated at birth is also the BRN and there is no need to transmit the UIN to another agency. This is the best-case scenario.

Electronic on-site birth registration in Botswana, for example, illustrates a process that assigns a UIN at birth within the same department that manages both civil registration and national identification. Assistant Registrars are stationed at major hospitals in Botswana. Soon after a newborn is delivered, the attending midwife or doctor completes a birth notification form and gives it to the Assistant Registrar who enters the information into an online registration system. The mother or father checks the accuracy of the information that is entered electronically, and then a UIN is generated from the central database. A birth certificate with the UIN listed on it is printed and given to the mother prior to discharge from the hospital. Subsequently, the UIN is used for the national identity card that is issued to an individual at age 16 years or above. In health centers and in small hospitals that do not provide on-site birth registration, a midwife or doctor completes the birth notification form, and within a week, the form is forwarded to the nearest registration office where the data are entered into the central database, quality-checked, and authorized by a supervisor. Since the information is captured in electronic form, the parent can go to any registration office in the country to obtain a printed copy of the birth certificate. However, the parent will have to show the relevant torn-off portion from the birth notification form and identify him- or herself.

Some countries assign the UIN randomly, while in other countries, it is logic-based (that is, data are based on location, birthdate, and sex). Norway and South Korea are examples of countries that use a logic-based UIN. Norway has an 11-digit identity number assigned at birth—the first 6 digits represent date of birth, the next two are individual numbers, the following number indicates sex (even numbers for women, odd numbers for men), and the last two are check digits (for control). South Korea has a 13-digit identity number assigned at birth—the first 6 digits represent date of birth, followed by 1 digit for gender, 4 digits for area code, 1 digit for the registered serial number, and 1 digit for the verification number. In contrast, India employs a random Aadhaar number, which has 12 digits (11 + 1 check digit). In the compendium of good practices in identity management, the Organization for Security and Co-operation in Europe indicated that the logic-based UIN reveals personal information and can facilitate data security breaches [8].

UINs can be generated online or offline [7]. The Botswana example above illustrates the approach to online assignment of a UIN. The offline approach comes into play in remote areas without Internet connectivity. For instance, countries could use a centrally pre-generated machine-readable sticker which is distributed to civil registration offices. A sticker with the pre-generated number is affixed to the registration form and a duplicate is sent to the central office to be entered in the database. Another offline approach is for all birth registrations to be done at a local office and information transmitted to the central office, where a UIN is generated and printed on the birth certificate and then the birth certificate is sent to the local office for the parent to pick up.

In such cases when there is linkage of civil registration and national identification systems, appropriate laws and other measures are necessary that require maintaining data confidentiality, privacy, and cyber security. Although the systems are linkable in principle, in practice, access is determined on a “need to know” basis; one agency does not need to have access to all data nor do all data need to be accessible from all systems.

## Beyond CRVS: the population register and UIN can contribute toward achieving UHC

Having universal and continuous data on vital events through CRVS has its utility for not only measuring SDG indicators, as explained above, but it also contributes to achieving SDG Target 3.8 (UHC). CRVS is foundational to achieving this target by counting everyone and making everyone visible with a legal identity. However, CRVS also has some limitations in this regard. While complete CRVS systems provide accurate numbers of live births and deaths, there are some other data that are not included as part of CRVS. For instance, data on migration are not collected in CRVS systems but are needed to count the actual number of people living in a given area and to more accurately compute the denominators for SDG indicators that require population values. As an example, to compute the proportion of 1-year-old children immunized against measles, the denominator (number of 1-year-old children) requires knowing the number of live births, the number of deaths, and net migration. Thus, CRVS could be strengthened by connecting to a database with data on migration. This underscores the importance of population registers. In countries where individuals are assigned UINs [9], a population register can link these individuals’ CRVS data with data from other functional registers (for example, registers for migration, health care, taxation, or schools) to create a broader network of reliable, up-to-date data pertaining to its population. Because civil registration registers deaths, the UIN allows the updating of associated registers (including health care) when a person dies. This linkage could be helpful for calculating service capacity and strengthening access to essential health services, including enrolling in a health insurance plan and identifying the most disadvantaged population. Standalone national identification systems neither provide evidence of family relationships and civil status, nor statistics on fertility and mortality. Similarly, standalone civil registration systems will not allow computation of population values, which are necessary for estimating some health indicators. Hence, coordination between the health sector, civil registration, national statistics office, immigration, and national identification systems with the use of the UIN, toward creating a population register, can be most effective.

## Conclusion

By assigning unique identification numbers to individuals, such as at birth registration, then using these numbers to link the individuals’ data from civil registration, national identification, and other functional registers, including registers for migration and health care, more accurate and disaggregated population values can be obtained. This is also key to improving the effectiveness of and access to social services such as education, health, social welfare, and financial services. When civil registration system in a country is linked with its national identification system, it benefits both the government and its citizens. For the government, having reliable and up-to-date vital events information on its citizens supports making informed program and policy decisions, ensuring the accurate use of funds and monitoring of development initiative performance at all levels. For individuals, it makes it easier to prove one’s identity and the occurrence of vital events to claim public services such as survivor benefits or child grants.

There are several ministries and institutions that collaborate closely for the functioning of these linked systems. Establishing clear institutional arrangements, such as the establishment of a multisectoral national CRVS coordination committee consisting of representatives from key stakeholder groups, is important and can facilitate the functioning of these linked systems, contributing to improving the effectiveness and efficiency of health service delivery, health coverage programs, and achievement of UHC [10].

## Data Availability

Not applicable

## Abbreviations

BRN: birth registration number
CRVS: civil registration and vital statistics
MMR: maternal mortality ratio
SDG: Sustainable Development Goals
UHC: universal health coverage
UIN: unique identification number
